# Eigenfaces-Based Steganography

**DOI:** 10.3390/e23030273

**Published:** 2021-02-25

**Authors:** Tomasz Hachaj, Katarzyna Koptyra, Marek R. Ogiela

**Affiliations:** 1 Institute of Computer Science, Pedagogical University of Krakow, 30-084 Krakow, Poland; 2 Cryptography and Cognitive Informatics Laboratory, AGH University of Science and Technology, 30-059 Krakow, Poland; kkoptyra@agh.edu.pl (K.K.); mogiela@agh.edu.pl (M.R.O.)

**Keywords:** steganography, eigenfaces, linear combination, principal components analysis, Log-Euclidean Distance

## Abstract

In this paper we propose a novel transform domain steganography technique—hiding a message in components of linear combination of high order eigenfaces vectors. By high order we mean eigenvectors responsible for dimensions with low amount of overall image variance, which are usually related to high-frequency parameters of image (details). The study found that when the method was trained on large enough data sets, image quality was nearly unaffected by modification of some linear combination coefficients used as PCA-based features. The proposed method is only limited to facial images, but in the era of overwhelming influence of social media, hundreds of thousands of selfies uploaded every day to social networks do not arouse any suspicion as a potential steganography communication channel. From our best knowledge there is no description of any popular steganography method that utilizes eigenfaces image domain. Due to this fact we have performed extensive evaluation of our method using at least 200,000 facial images for training and robustness evaluation of proposed approach. The obtained results are very promising. What is more, our numerical comparison with other state-of-the-art algorithms proved that eigenfaces-based steganography is among most robust methods against compression attack. The proposed research can be reproduced because we use publicly accessible data set and our implementation can be downloaded.

## 1. Introduction

Steganography is a technique of hidden communication. The fact of passing messages between sender and recipient is kept secret by embedding messages in inconspicuous containers. These may be either common files, for example images and videos, or unexpected media, like geospatial data [1], network packets [2] and others [3]. In the digital era nearly any type of file may carry additional hidden data, but images are among most popular because the human visual system is unable to perceive subtle changes introduced by embedding process. There are many possible approaches to image-based steganography and we will discuss selected methods in the following subsection.

### 1.1. State-of-the-Art in Steganography

In spatial domain, the most known steganography technique is least significant bit (LSB) in which some bits of pixels are replaced with bits of a message. It may utilize various numbers of bits and mapping strategies, for example in [4,5] four least significant bits are substituted. Algorithm described in [6] uses plane bit substitution method in which message bits are embedded into the pixel value(s) of an image. A steganography transformation machine is proposed for solving binary operation for manipulation of the original image with help of LSB operator based matching. Ref. [7] uses five pixels pair differencing technique that is a combination of LSB and pixel value differencing (PVD) steganography. In [8] LSB message encoding is preceded by CRC-32 checksum, then the codeword is compressed by gzip just before encrypting it by AES, and is finally added to encrypted header information for further processing. During embedding the encrypted data, the Fisher–Yates Shuffle algorithm is used for selecting the next pixel location.

Least significant bit replacement is among the most used spatial domain techniques whereas discrete cosines transform (DCT) [9] and discrete wavelet transform (DWT) [10] based methods are major choices in the frequency domain. Additionally, many steganography techniques are based on singular value decomposition (SVD) for embedding secret message. These techniques conceal data in either right singular vectors, left singular vectors, singular values or combinations of all approaches in spatial domain or transform domain with satisfactory performance to various attacks [11,12,13,14]. Paper [15] describes image and [16] video steganography for face recognition for trusted and secured authentication by applying principal component analysis (PCA), namely the eigenfaces method. In those approaches signcryption is included for additional security measures.

An opposite approach for image steganography is presented in paper [17] where authors presented new generating technique which does not require any additional image to cover secret text. Instead, the image is created from scratch, on the basis of the text that we want to send securely.

The other steganographic techniques are based on the key generating process. In paper [18] authors used a new method of data hiding using Catalan numbers and Dyck words. The hidden message is generated with the data carrier and an adequate complex stego key. An important characteristic of the proposed method is that the data carrier retains its original shape, without supplements or modifications. Paper [19] presents an effective method of encryption and decryption of images in multi-party communications. Encryption is based on weighted Moore–Penrose inverse over the constant matrix.

There are many documents describing state-of-the-art steganography methods. A comprehensive discussion about combining individual’s biometric characteristics with steganography may be found in paper [20]. The more general surveys are presented in [21,22,23,24].

### 1.2. Motivation of This Paper

In this paper we propose a novel transform domain technique in which the message is hidden in components of linear combination of high order eigenfaces vectors. By high order we mean eigenvectors responsible for dimensions with low amount of overall image variance, which are usually related to high-frequency parameters of image (details). They seem to have marginal influence on image quality, especially if the eigenfaces method is trained on large enough data sets.

The choice of facial images as medium is based on overwhelming influence of social media in which hundreds of thousands of photos are uploaded every day. The high percentage of them are selfies which do not arouse any suspicion as potential steganography communication channel.

The main difference between proposed algorithm and already existing solutions is that our method utilized a certain fragments of the image, namely faces that are present in pictures. Due to this fact after hiding a secret, the rest of the image beside faces remains unmodified. Thanks to this there are no global histogram shifts in the image, which is a main indicator of potential image modification. What is more, we wanted our method to be highly robust to various common image transformations. We wanted obtain a bit-level accuracy of encoded data rather than pixel-level accuracy like in [14], so our priority was robustness rather than carrier capacity. Therefore proposed method can be easily used not only to watermarking but also to communication through the channel that does not preserve integrity of the message, for example when the image is uploaded on social media, its quality may be altered.

For our best knowledge there is no description of any popular steganography method that utilizes eigenfaces image domain. Due to this fact we have performed extensive evaluation of our method using at least 200,000 facial images for training and robustness evaluation of the proposed approach. The proposed research can be reproduced because we use publicly accessible data set and our implementation can be download from GitHub repository (https://github.com/browarsoftware/EigenfacesSteganography, accessed on 30 January 2021).

The paper is composed of five sections. In the Section 2 we have presented proposition of our steganography method, the dataset we have used for tests and evaluation metrics. We have performed extensive validation of proposed method which results are presented in Section 3. In the Section 4 and Section 5 and we have discussed obtained results presenting advantages and disadvantages of eigenfaces-based steganography and potential solutions that can be used to overcome its drawbacks.

## 2. Materials and Methods

In this section we propose our novel method, present the data set we used for training and validation and explain robustness tests we have done.

### 2.1. Eigenfaces and Eigenfaces-Based Steganography

An eigenface is a *k*-dimensional vector of real values that represents features of pre-aligned facial image. Eigenfaces are based on principal component analysis (PCA). Let us suppose that we have a set of *l* images $\left[I_{1},I_{2},\ldots ,I_{l}\right]$ with uniform dimensionality m×n. Each face image being initially a two-dimensional matrix is “flattened” by placing its columns one below another, thereby becoming a single column vector. Then we use PCA to perform variance analysis and to recalculate current coordinates of those vectors into PCA coordinates systems where axes are ordered from those that represent highest variance to those that represent lowest variance. Let us assume that we have matrix *D* with dimensionality (n·m)×l in which each column is a flattened image:

(1) $$D_{\left[n\cdot m,l\right]}=\left[I_{1},I_{2},\ldots ,I_{l}\right]$$

Next we calculate a column vector *M* (so called mean face) in which each row is a mean value of a corresponding row in matrix *D*

(2) $$M=\left[\begin{array}{c} \sum_{i=0}^{l}\frac{I_{i,1}}{l}\\ \sum_{i=0}^{l}\frac{I_{i,2}}{l}\\ \vdots \\ \sum_{i=0}^{l}\frac{I_{i,n\cdot m}}{l} \end{array}\right]$$

where $I_{i,j}$ is the *j*-th pixel of *i*-th image. This mean face is then subtracted from each column of matrix *D* to calculate new matrix called $D^{\prime }$:

(3) $$D_{\left[n\cdot m,l\right]}^{\prime }=\left[I_{1}-M,I_{2}-M,\ldots ,I_{l}-M\right]$$

Then a covariance matrix is created:

(4) $$C_{\left[l,l\right]}=\frac{1}{l}\cdot D_{\left[n\cdot m,l\right]}^{\prime T}\cdot D_{\left[n\cdot m,l\right]}^{\prime }$$

Because *C* is symmetric and positively defined, all eigenvalues have real positive values. Eigenfaces *E* are calculated as:

(5) $$E_{\left[n\cdot m,l\right]}=D_{\left[n\cdot m,l\right]}^{\prime }\cdot E_{C[l,l]}$$

where $E_{C}$ are eigenvectors of *C* ordered from the highest to the lowest eigenvalue.

In order to generate eigenfaces-based features $e_{i}$ of face image $I_{i}$ one has to perform following operation:

(6) $$e_{i}=E_{\left[n\cdot m,l\right]}^{T}\cdot \left(I_{i}-M\right)$$

The inverse procedure that recalculates image vector coordinates to original coordinate system is:

(7) $$I_{i}^{\prime }=\left(E_{\left[n\cdot m,l\right]}\cdot e_{i}\right)+M$$

We can use *k* first eigenvectors where k<l. In this case $(I_{i}\approx I_{i}^{\prime }$ and $I_{i}^{\prime }$ keeps at least percentage of variance equals to scaled cumulative sum of eigenvalues cooresponding to *k* first eigenvectors. In other words when k<l, vector $I_{i}^{\prime }$ represents “compressed” facial image $I_{i}$ in respect to overall variance. $e_{i}$ features are coefficients of linear combination of *E*. Coefficients with lower indices corresponds to dimensions with higher variance.

Eigenface-based feature calculation is performed after designation of $E_{\left[n\cdot m,l\right]}$ in (5). We have to subtract mean vector *M* from the face image $I_{i}$ in the same manner, as it was performed in (3). Image $I_{i}$ may be replaced by any face image, also not included in *D*, however it has to have the same resolution as images in *D*, namely m×n. When $I_{i}$ is not from data set *D*, then we can safely assume that $I_{i}\approx I_{i}^{\prime }$.

The idea of eigenface-based steganography is to replace range of coefficients $e_{\left[j,j+o\right]}$ with a binary encoded message *s* with the length *o*. *j* is an offset from the beginning of the eigenface-based features vector. The message is stored by changing original values of linear combination to binary values: $\left\{\frac{-1}{div},\frac{1}{div}\right\}$ where $\frac{-1}{div}$ represents 0, $\frac{1}{div}$ represents 1 and div is a scaling parameter. Transformation of original binary message *s* into values of message $s^{\prime }$, which is directly inserted into eigenfaces coefficients, goes as follows:

(8) $$s_{i}^{\prime }=\frac{\left(2\cdot s_{i}-1\right)}{div}$$

where $s_{i}$ is *i*-th binary coefficient of vector *s* that contains a message to hide. The inverse procedure is:

(9) $$s_{i}=\mathrm{round}\left(\frac{s_{i}^{\prime }\cdot div+1}{2}\right)$$

where round denotes rounding to the closest integer.

**Figure 1:**
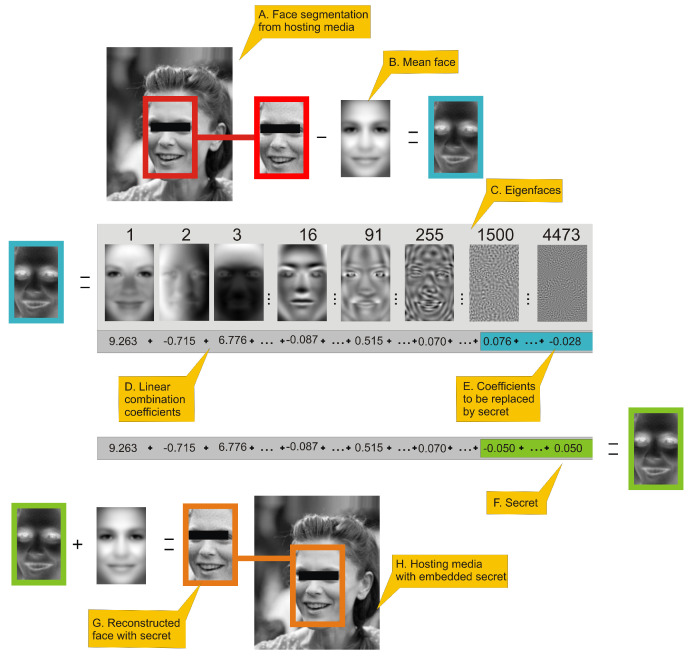
This figure presents an outline of eigenface-based steganography secret encoding framework. (**A**.) a face image is extracted from an image; (**B**.) a mean face is subtracted from face image. (**C**.) presents selected eigenfaces visualized as 2D images. (**D**.) By applying Equation (6) we can generate eigenfaces-based features of a face, which is a linear combination of coefficients. Values in sum (**D**.) are actual coefficients of the face in turquoise frame. High-order coefficients (**E**.) of that linear combination have fractional influence on reconstructed image visual quality and can be used to hide the secret. Hiding can be done simply be replacing those coefficients by rescaled secret with Equation (8). (**F**.) is a linear combination coefficient with high-order coefficients replaced by a secret. That linear combination is used to reconstruct face image using Equation (7), which is represented as the face inside green frame. The last two steps is: (**G**.) adding mean face to face with hidden data and: (**H**.) inserting face image to original hosting media.

**Figure 2:**
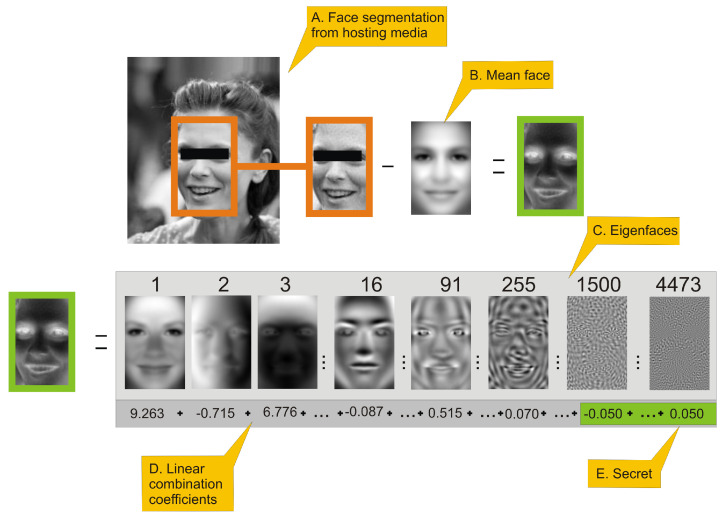
This figure presents an outline of eigenfaces-based steganography secret decoding framework. The person in the image has been anonymized. (**A**.) a face image is extracted from an image with embedded secret; (**B**.) a mean face is subtracted from face image. (**C**.) presents selected eigenfaces visualized as 2D images. The number above eigenface is its index (those are same eigenfaces as in Figure 1). (**D**.) By applying Equation (6) we can generate eigenface-based features of a face, which is a linear combination of coefficients (**D**.) Values in sum (**D**.) are actual coefficients of the face in orange frame. High-order coefficients (**E**.) of that linear combination are secret data scaled by Equation (8). In order to recover original data we have to apply Equation (9) to those coefficients.

The offset value *j* and the maximum value of secret length *o* need to be determined. It is a subject of discussion in following sections. The message hiding algorithm is presented in Algorithm 1. Message recovering algorithm is presented in Algorithm 2.
 **Algorithm 1:** Message encoding algorithm.  **Data**: *J*—image containing a face,  *s*—message to hide with length *o* bits,  *j*—offset from which the coefficients will be replaced by message,  div—divider parameter.  **Result**: Image $J^{\prime }$ with hidden data.  1. Extract face image from *J* (Figure 1A);  2. Perform aligning of extracted face and store it in matrix $I_{i}$;  3. Generate $e_{i}$ from $I_{i}$ using (6) (Figure 1B,D);  4. Starting from index *j* replace original *o* values in $e_{i}$ by $s_{i}^{\prime }$, which are scaled coefficients that represents binary message data (8) (Figure 1E,F);  5. By application of (7) generate $I_{i}^{\prime }$ using modified $e_{i}$ (Figure 1G);  6. Insert $I_{i}^{\prime }$ into *J* replacing original face, $J^{\prime }$ image is created (Figure 1H);

Figure 1 presents the procedure of encoding a secret. All numerical values presented in this figure will be justified in the Section 3. Person in image has been anonymized. The number above eigenface is its index. Eigenfaces are ordered by decreasing eigenvalues that corresponds to them. As can be seen several first eigenfaces represents global properties of image (i.e., lighting), next eigenfaces are responsible for high-frequency details.
 **Algorithm 2:** Message decoding algorithm.  **Data**: *J*—image containing a face,  *j*—offset from which the coefficients have been replaced by message,  div—divider parameter  **Result**: *s*—decoded message with length *o* bits.  1. Extract face image from *J*;  2. Perform aligning of extracted face and store it in matrix $I_{i}$;
  3. Generate $e_{i}$ from $I_{i}$ using (6);
  4. Starting from index *j* extract *o* values from $e_{i}$ which are rescaled message’s values ($s_{i}^{\prime }$), then restore the binary message $s_{i}$ by rescaling with div parameter (9);

The procedure of decoding a secret, which is presented in Figure 2, is very similar to process of encoding.

### 2.2. The Data Set

In order to successfully generate eigenfaces with a strong descriptive power and to validate our approach, we needed a sufficiently large collection of faces. Among the largest publicly available open faces repositories we have chosen Large-scale CelebFaces Attributes (CelebA) Dataset [25] which may be downloaded from Internet (http://mmlab.ie.cuhk.edu.hk/projects/CelebA.html, accessed on 1 January 2021). It consisted of 202,598 images of celebrities, actors, sportspersons etc. In this research we have used aligned and cropped version of those images. Each of aligned and cropped photos has the same resolution and the face is centered so that eyes of each person are located nearly in the same position. There is no information how the alignment has been done; however it can be very closely reproduced with Histogram of Oriented Gradients (HOG) method [26]. The face position estimator can be created using Python dlib’s library implementation of the work of Kazemi et al. [27] with face landmark data set (https://github.com/davisking/dlib-models, accessed on 1 January 2021.) trained on [28]. In order to reduce the complexity of further computations, we have limited the size of images to resolution 70×109, cropping all regions of image beside very face. For the same reason we have also converted images from RGB to grayscale. We have to remember, however, that proposed steganography approach may also be applied to RGB data, because eigenfaces are calculated in the same way on multiple colour channels as on single channel. Nonetheless, this discussion is out of scope of this paper.

### 2.3. Comparison of Covariance Matrices

The very heart of eigenfaces approach is preparation of a representative data set that is used to calculate linear transform from original coordinates to space (obtained by principal component analysis). Theoretically the more face samples we take (provided that the data set covers representative sample of images for future encoding), the better variance analysis we get. However, given that we operate on personal computers with limited RAM, we have to keep in mind that solving eigenvalue problem is complex and time demanding task, especially for relatively large matrices (i.e., $10^{4}\times 10^{4}$ or larger). Due to this we wanted to estimate the size of training set above which the covariance matrix will not change much. In order to do so, we compared the geodesic distance between covariance matrix generated from whole training data set $C_{\mathrm{ref}}^{2}$ and covariance matrices generated from subsets of validation data set of different sizes $\left[C_{k_{1}}^{2},\ldots ,C_{k_{p}}^{2}\right]$ where $\left[k_{1},\ldots ,k_{p}\right]$ are subsets of validation data set.

In order to calculate covariance matrix $C^{2}$ we used formula:

(10) $$C_{\left[n\cdot m,n\cdot m\right]}^{2}=\frac{1}{l}\cdot D_{\left[n\cdot m,l\right]}^{\prime }\cdot D_{\left[n\cdot m,l\right]}^{\prime T}$$

where $C^{2}$ has the same dimension [n·m,n·m] no matter how many images *l* were used in calculation.

Due to the fact that covariance matrices are symmetric positive definite, we can measure distance between them using the Log-Euclidean Distance (LED) [29]. Let as assume that *A* and *B* are symmetric positive definite. The geodesic distance between *A* and *B* can be expressed in the domain of matrix logarithms [30] as:

(11) $$LED\left(A,B\right)={∥\log(A)-\log(B)∥}_{F}$$

In the above equation ${∥∥}_{F}$ is a Frobenius norm which is calculated for *n* by *m* matrix *A* as [31]:

(12) $${∥A∥}_{F}=\sqrt{\sum_{i=1}^{n}\sum_{j=1}^{m}{\left|a_{ij}\right|}^{2}}$$

Let us assume that *C* and *D* are square matrices. A matrix *C* is a logarithm of *D* when:

(13) $$e^{C}=D$$

where matrix exponential is defined as:

(14) $$e^{A}=\sum_{i=0}^{\infty }\frac{A^{i}}{i!}$$

Any non-singular matrix has infinitely many logarithms. As the covariance matrix does not have negative eigenvalues, we can use method described in [32] to calculate the logarithm.

Another approach for calculating influence of correlation matrix size on quality of reconstruction from limited number of PCA coefficients is based on two measures: averaged mean square error (MSE) (15) between actual (Ac) and reconstructed (Re) image and averaged Pearson correlation coefficient (CC) (16) between actual and PCA compressed image. Mean square error between actual and reconstructed image is defined as:

(15) $$MSE\left(Ac,Re\right)=\frac{1}{n}\sum_{i=1}^{n}{\left(Ac_{i}-Re_{i}\right)}^{2}$$

The Pearson correlation coefficient (CC) between actual Ac and reconstructed Re image is defined as:

(16) $$CC\left(Ac,Re\right)=\frac{Cov(Ac,Re)}{\sigma_{Ac}\sigma_{Re}}$$

where Cov(Ac,Re) is covariance coefficient between Ac and Re and $\sigma_{Ac}$, $\sigma_{Re}$ are standard deviations of Ac and Re.

To better visualize relationship of LED and size of covariance matrix, we can also use relative values of LED coefficient:

(17) $$\mathrm{Relative}\;LED_{i}=\frac{LED_{i}-LED_{i-1}}{LED_{i}}$$

Relative MSE and Relative CC may be calculated similarly as in (17).

LED geodesic distances were compared with averaged mean square error (MSE) and averaged Pearson correlation coefficient (CC) between actual and reconstructed images from validation data set generated by PCA. The goal of this test was to check if the geodesic distance corresponds to averaged MSE and CC of PCA-compressed data.

### 2.4. Robustness Tests

We tested the robustness of proposed steganography method to common transformations. Each test has been applied to the image that contained hidden data.

- Rotation of image about its centre element using third-order spline interpolation.
- Salt and pepper—replacing given number of randomly chosen pixels with either 0 (black) or 255 (white) value.
- JPEG compression with various quality settings [33].
- Linear scaling with bicubic pixels interpolation.
- Image cropping—for obvious reasons the eigenfaces method is very sensitive to image cropping, however it seems to be resistant to mixing encoded signal with an original image. We can do it with following steps. At first we create a matrix Cir, which elements $cir_{ij}$ are defined using following formulas:
  (18) $$cir_{ij}={\left(\frac{2\cdot i}{n}-1\right)}^{2}+{\left(\frac{2\cdot j}{m}-1\right)}^{2};\;i\in 0,\ldots ,n;\;j\in 0,\ldots ,m$$
  (19) $$Cir=1-\frac{1}{2}\left[\begin{array}{ccc} cir_{1,1} & \cdots & cir_{1,m}\\ \vdots & \ddots & \vdots \\ cir_{n,1} & \cdots & cir_{n,m} \end{array}\right]$$
  We can use that matrix to mix the original image *I* with image with encoded data *E* by linearly scaling the amount of *E* using threshold parameter *t*:
  (20) $$Cir_{t}=\left\{\begin{array}{ccc} a_{i,j} & when & a_{i,j}<1-t\\ 1 & when & a_{i,j}\geq 1-t \end{array}\right.$$
  (21) $$Mix=Cir_{t}\cdot E+\left(1-Cir_{t}\right)\cdot I$$

**Figure 3:**
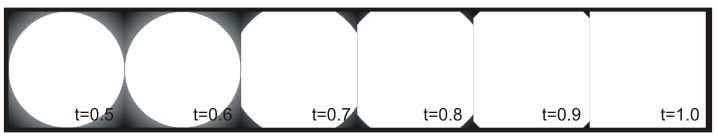
This figure presents grayscale-coded shapes of circular elements defined in Equations (17)–(19) depending of value of threshold *t*.

Figure 3 presents how changes the shape of circular elements when the value of threshold *t* increases.

Messages retrieved from disturbed images were compared with the original message using following similarity coefficients or measures:

- Binary coefficient (BC) that equals 0 when both messages are identical and 1 otherwise.
- Levenshtein distance (LD) [34] which is a measure of the similarity between two strings. The distance is the number of deletions, insertions, or substitutions required to transform one into another. The greater the Levenshtein distance, the more divergent those strings are [35].
- Sørensen–Dice coefficient [36]:
  (22) $$DSC\left(v_{1},v_{2}\right)=\frac{2\cdot (1-\left|v_{1}\cap v_{2}\right|)}{\left|v_{1}\right|+\left|v_{2}\right|}$$
  where $v_{1}$, $v_{2}$ are binary vectors and $\left|v\right|$ is cardinal of binary vector *v*.

We have also used two following metrics to compare results obtained by our solution to state-of-the-art method: Pearson correlation (CC) and peak-signal-to-noise-ratio (PSNR). CC is used to evaluate the similarity between the original secret and the secret message and PSNR in dB is used to evaluate the similarity of the original image and the stegoimage.

(23) $$PSNR=10\cdot log_{10}\frac{l_{max}^{2}}{MSE}$$

where $l_{max}^{2}$ is the maximum value of vector pixels over the original image (in our case 255) and the MSE is represents the mean square error between the stegoimage and the original image.

### 2.5. Hiding Data in Larger Images

When the facial image is a part of larger photo (which is true in most cases), it should first be extracted, then encoded and decoded using eigenfaces and finally inserted back into the photo. As the modified face is not identical to the original, it may be possible to spot visual artifacts in a rectangular region containing a face. In order to blur the manipulation, we can use the clipping procedure described in Equations (18)–(21). Then, step 5 of the message encoding algorithm (Algorithm 1) is extended in following manner:

 

5. By application of (7) generate $I_{i}^{\prime }$ using (8), apply (18)–(21) to blur borders of inserted data.

 

The algorithm has one additional parameter *t* (threshold from (20)). The message decoding algorithm (Algorithm 2) remains unchanged.

## 3. Results

The proposed solution was implemented in Python 3.8 (however it seemed there were no obstacles to run it on lower version of this language). Among the most important packages we used was OpenCV-python 4.1.2.30 for general purpose image processing. For algorithms training and evaluation we used a PC computer equipped with Intel i7-9700F 3.00 GHz CP, 64 GB RAM, NVIDIA GeForce RTX 2060 GPU with Windows 10 OS, and the second PC with similar hardware architecture, however with 128 GB RAM with Linux OS.

We used data set described in Section 2.2 to evaluate this research. The faces data set was divided into halves. The first half (101,299 faces) was used as the training data set; the second half (101,300 faces) was a validation/test data set.

At first we estimated how many images we should use to create eigenfaces. In order to do so, we compared covariance matrices using methodology described in Section 2.3. To create $C_{\mathrm{ref}}^{2}$ (9) matrix, we used the whole validation dataset. In order to create $C_{k_{a}}^{2}$ matrices, we used training dataset that was divided into subsets. Subset with index *a* contained $\#k_{a}=2025\cdot a$ faces (2025 is about 2% of training dataset), where a∈[1,2,…,p].

$C_{\mathrm{ref}}^{2}$ and $C_{k_{a}}^{2}$ were compared using Log-Euclidean Distance (10). It was possible because both matrices had identical dimensions. Then, for each $C_{k_{a}}^{2}$, every image $I_{b}$ in training data set was encoded and then decoded using eigenfaces that explained at least 0.999 of variance; as a result an image $I_{b}^{\prime }$ was created. MSE (15) and CC (16) were calculated for $I_{b}$ and corresponding $I_{b}^{\prime }$ (they were calculated for images from the test data set because $C_{k_{a}}^{2}$ were generated from validation data set). Averaged values of MSE and CC showed how well eigenfaces of various $C_{k_{a}}$ described the data set. We also made calculations of relative values of LED and MSE according to Equation (16). CC coefficient had a high value from the beginning and did not change much and due to this we skipped calculation of relative CC. When we used 30,375 images to create correlation matrix for PCA, both relative LED and relative MSE dropped below 0.03. Basing on this fact, we decided that it might be a sufficient size of the training data set to evaluate our methodology. What is more, calculation of eigen decomposition for $3\times 10^{4}$ matrix could be done in a reasonable time and further increase of data set did not change much in LED and averaged MSE. We have marked our choice of dataset size in Table 1 with two horizontal lines.

**Figure 4:**
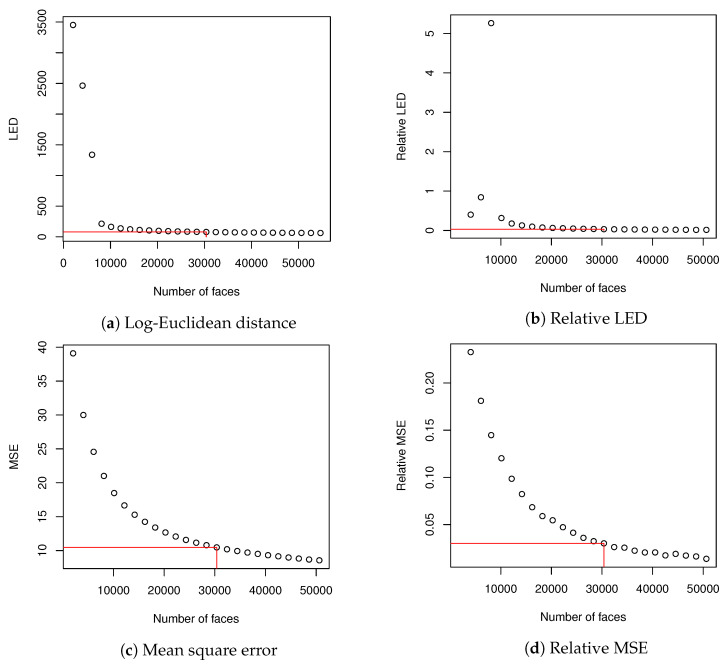
This plot visualizes data from Table 1. Red line indicates our choice of number of faces in training data set that was later used to generate eigenfaces in second part of the experiment. It was justified in Section 3.

Results from Table 1 (beside CC, which did not change much during the experiment) are visualized in Figure 4.

There was a limited amount of data to be hidden within eigenfaces, which were constrained the most by the face image resolution that was used to produce matrix *D* (1) and then $E_{\left[n\cdot m,l\right]}$ (5). The second constraint was distribution of variance among eigenfaces, the influence of which on the capacity of the medium is discussed later on.

Typically steganography algorithms are evaluated on set of benchmark images like Lena, Pepper, Airplane etc. In our case however eigenfaces-based steganography operates only on face data. Because of it we need different validation dataset. Evaluation of robustness of proposed steganography algorithm was performed with methodology described in Section 2.4. We used training data set containing 30,375 faces and validation data set with 101,300 faces. Scaling parameter div in binary data encoding was arbitrary set to 20.

After applying PCA, we have calculated the number of dimensions that describe variance in test data set (consisting of 30,375 faces). 0.75 of variance was explained by 15 dimensions, 0.9 variance by 90 dimensions, 0.95 variance by 254 dimensions, 0.99 variance by 1499 dimensions and finally 0.999 variance by 4473 dimensions. Larger number of dimensions used for image encoding may introduce some high-frequency noises caused by not statistically significant data fluctuations in training data set. We can encode data in any eigenfaces coefficients between first and 4473rd value; however changes in linear combination of coefficients that are more important for variance explanation will be clearly visible in the encoded image. Because of that we decided to modify data between 1499 and 4473 coefficients. In this configuration potential maximal capacity of the image is (4473−1499)/8≈371 Bytes. In further tests we have considered following lengths of messages: 18 bytes (∼5% of capacity), 37 bytes (∼10% of capacity), 87 bytes (∼23% of capacity), 174 (∼47% of capacity) and 370 (∼100% of capacity). We have chosen those values for convenience of calculations; also distribution of lengths allows to nicely plot the dependencies of proposed steganography method performance as a function of robustness tests parameters. Using five possible values of messages length unevenly distributed in data set allows visualizing general characteristics of proposed method. Of course it is possible to evaluate method using more sample points, however it would not introduce much new information. Additionally, evaluation of such large validation data set (approximately 100,000 images) lasted 24 to even 36+ h for each robustness test on hardware that we have used.

**Figure 5:**
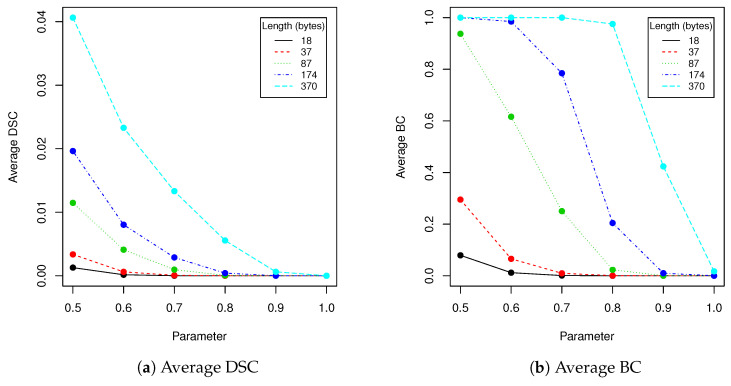

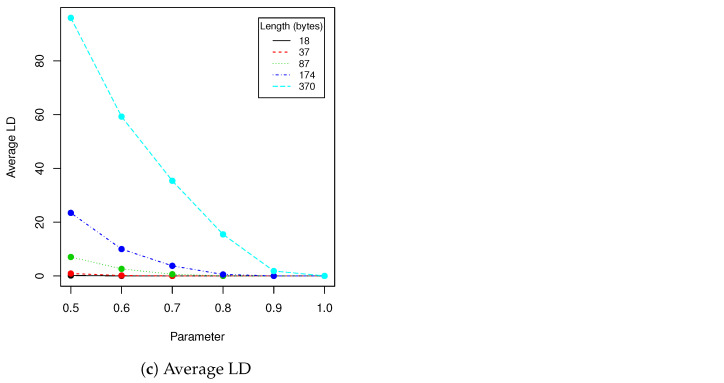
This figure presents visualization of data from Table 2 (clipping).

**Figure 6:**
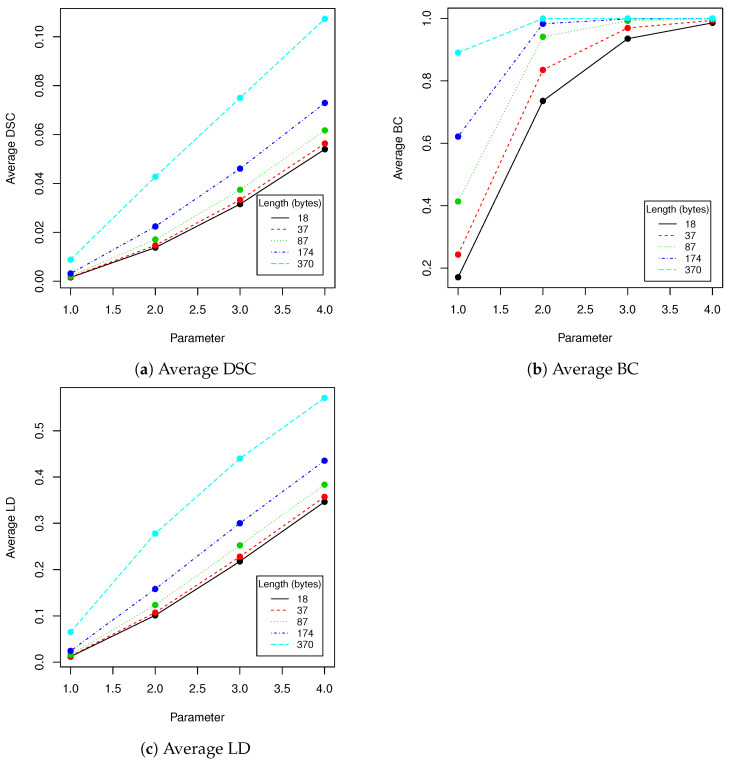
This figure presents visualization of data from Table 3 (salt and pepper).

**Figure 7:**
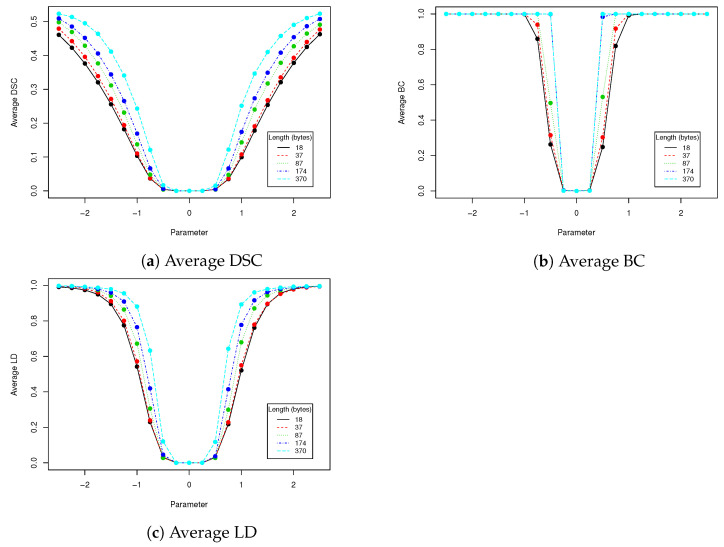
This figure presents visualization of data from Table 4 (rotation).

**Figure 8:**
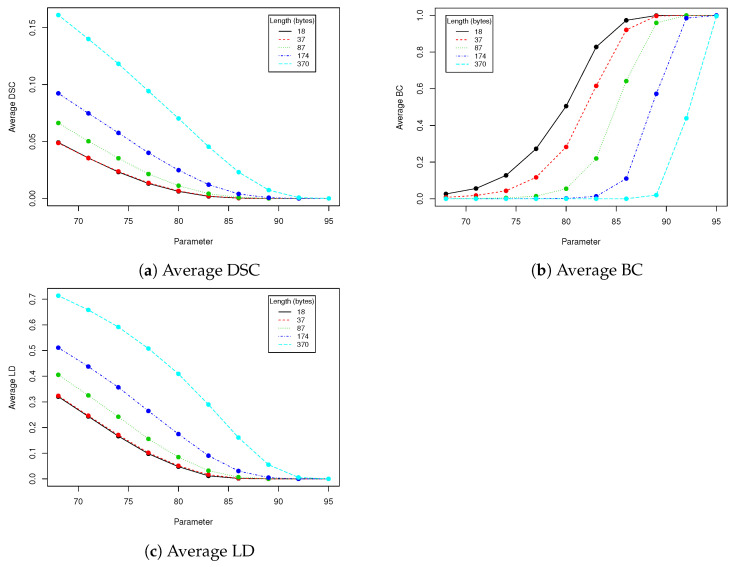
This figure presents visualization of data from Table 5 (JPEG compression).

**Figure 9:**
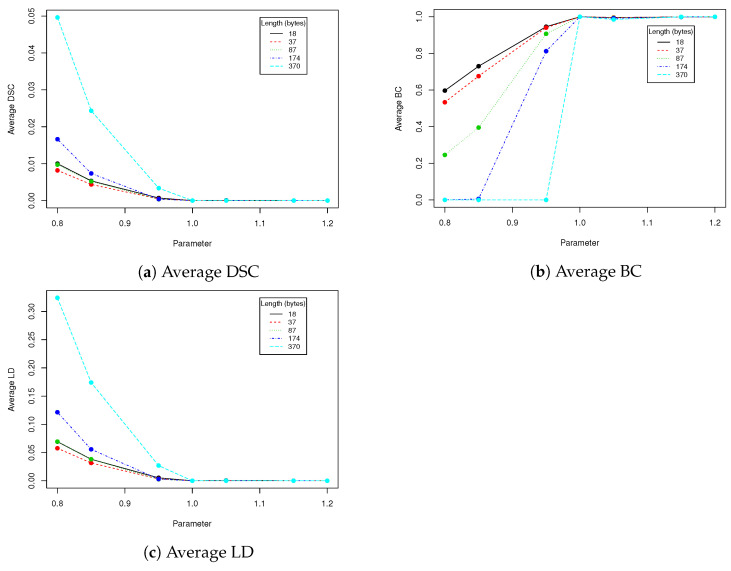
This figure presents visualization of data from Table 6 (scaling).

In robustness tests, besides various parameters described in Section 2.4, we used five mentioned lengths of encoded messages (18, 37, 87, 174 and 370). Obtained results are presented in Table 2, Table 3, Table 4, Table 5 and Table 6 and Figure 5, Figure 6, Figure 7, Figure 8 and Figure 9 using averaged values of Binary coefficient (BC), Levenshtein distance (LD) and Sørensen–Dice coefficient (DSC) on all images from validation data set.

**Figure 10:**
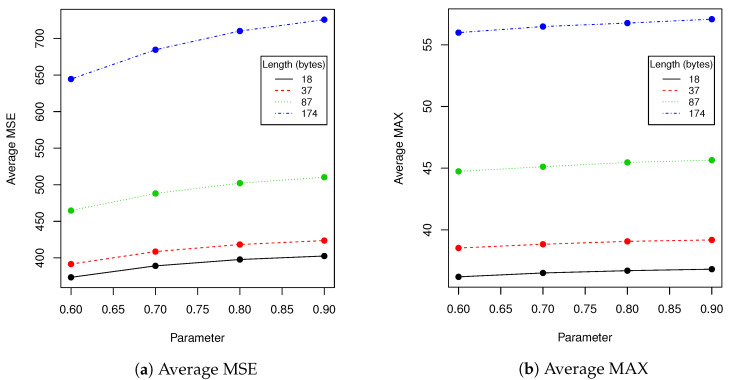

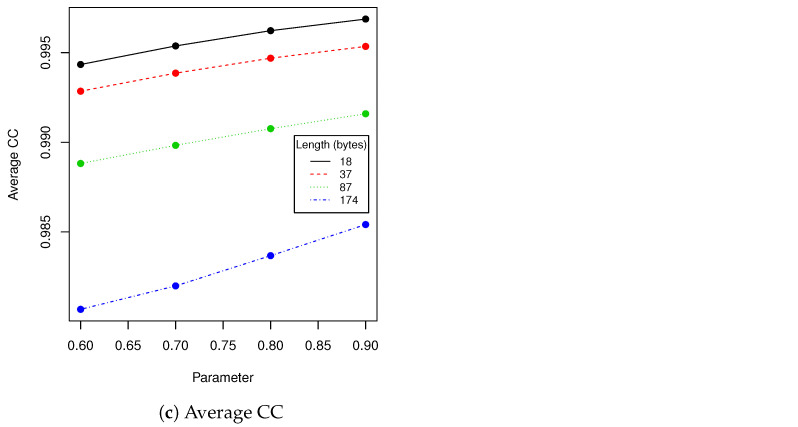
This figure presents visualization of data from Table 7 (prototype evaluation).

**Figure 11:**
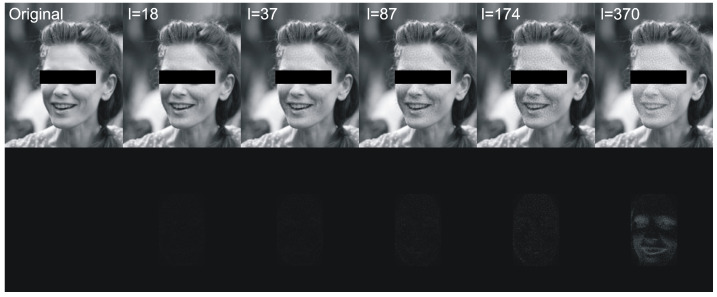
This figure shows an exemplary original image and images with hidden data of various lengths (first row) and differences between original image and images with hidden data of various lengths (bottom row). The threshold parameter for mixing (19) was set to t=0.6.

Next we have tested performance of algorithms described in Section 2.1 and Section 2.5 using various lengths of encoded messages and *t* parameter. We have calculated following statistics between original image and image with hidden data: averaged MSE, averaged maximal difference of pixels and averaged Pearson correlation coefficient of pixels. Results are presented in Table 7 and Figure 10. Figure 11 visualizes example differences between original image and image with hidden data. In some tables we have skipped certain zero-filled ranges in order to make results shorter and more comprehensible for reader. The larger ranges of values are presented in figures.

The proposed method has been compared with following contemporary algorithms [11,12,13,14] in terms of CC and PSNR—see Table 8. We have evaluated robustness of the proposed steganography method (PM) against compression attack, which seems to be most common scenario in case of publishing stego images in social media.

## 4. Discussion

For the reasons described in Section 3, we have selected training data set containing 30,375 faces to generate eigenfaces. This value might have been different if the calculation had been done on images with different resolution or on different data set, however the reasoning remains unchanged. In order to make eigenfaces descriptive enough for particular resolution, we need to take data set for which Relative LED (or/and Relative MSE) is below certain threshold. In our case 0.03 seems to be adequate value—as can be seen in Figure 2, it introduces plateau on the Relative MSE and Relative LED plots. Taking more faces to generate covariance matrix would not change much in obtained results. The rest of calculations and discussion is conducted on eigenfaces generated from selected data set.

The proposed steganography method exhibits differing resistance to robustness tests. Although it it quite vulnerable to salt and paper disturbance (see Table 3), it seems to deal very nice with changes applied to whole image domain. Both clipping and JPEG compression do not damage the message if the whole possible capacity is not exhausted. The study found that when message length is set to 87 bytes, we can safely use a quality coefficient equal to 89, which only affects 0.041 (4.1%) of messages (see Table 5).

When image quality equals to 92 or more, no changes are observed for all validation data set (containing more than 100,000 of various faces images!). Result for BC measure corresponds to DSC and LD coefficients. The longer the message becomes, the more errors are introduced by compression. This situation is very similar if we take into account clipping (see Table 2). When the message length is set to 37 bytes and t=0.6, the BC equals 0.066 which means that over 93% of messages have been successfully recovered. When *t* increases to 0.9, the successful recovery rate rises to 100%. However the larger *t* we take, the more visible is a rectangular region in which message is hidden. Due to this fact, when the message lenght is set to 37 (10% of image capacity), we suggest using *t* in range of [0.6,0.7] in order to increase difficulty of message detection. More details will be provided together with performance tests.

The limitation of the proposed method is basically the same as in the original eigenfaces approach [37]. The descriptive power of the method is determined by the variance of images in dataset *D* that was used to generate eigenfaces. There might be a face with a certain facial features which are not represented in D and reconstructed face $I_{i}^{\prime }$ might differ from the original face $I_{i}$. Those differences will be visible as high frequency noises and they might be easily spotted. The most straightforward solution to this issue is to use large dataset D with diversified face features. From the same reason face of the person wearing heavy, untypical makeup might be inappropriate carrier of hidden data. Additionally, it is recommended that a person on the photo should face the camera, which is an additional limitation.

Because rotation affects face aligning, eigenfaces are not robust to rotation (or robustness is on very low level—see Table 4). However this weakness may be compensated by face image aligning. The HOG-based face features detection, which is often used for this task, is a robust and repeatable technique that performs translation, rotation and scaling of original image. Due to this fact those three linear transformations, which might be used to change images with encoded data, might be then compensated by face image aligning. This, however, depends of face aligning procedure that we apply and it is not in the scope of this paper.

Eigenfaces technique requires that a facial image need to have the same resolution as faces in training dataset that was used to generate eigenfaces. We have to check if scaling the image with hidden data and then returning to the old resolution affects the payload, provided that we use bicubic pixels interpolation. Results in Table 6 show that our method has limited robustness for downscaling and very good robustness for upscaling. For upscaling by 5%, more than 98% messages were unaffected for all tested lengths; the more we upscale the better results we get. This means that we can use eigenfaces generated from images with lower resolution to hide data into facial images with higher resolution. A facial image has to be downscaled and then upscaled to old resolution. This is very important information, because thanks to that our method can be used in a certain range of facial images resolutions without necessity to generate eigenfaces to each possible resolution. Of course if we upscale image too much, a person observing the face might spot artifacts caused by interpolation.

The next experiment we have done was evaluation of the performance of the proposed algorithm with various encoded message lengths and *t* parameter (cropping size) values. Obtained results are presented in Table 7 and Figure 10. We have compared over 100,000 original images from the validation data set with images with encoded data using MSE, maximal difference between corresponding pixels and CC averaged. Those results confirmed observations we have made in robustness evaluation. Parameters in range [0.6,0.7] resulted in a relatively small value of distance between original image and one with encoded data. As can be seen in second row of Figure 11, when message size is set to 37 (10% of source image capacity) and t=0.6, the differences are virtually impossible to notice. Those parameters are our recommendation for this particular image resolution we have evaluated. After message encoding we need to check if it can be recovered (according to Table 2 in 6.6% faces there might be a problem with it). To overcome this situation, we have to increase *t* value or use another facial image. When message length is close to 50% of image capacity, we can even visually spot that some changes has been applied to the region of the face. We do not recommend exploiting full possible image capacity: it is better to split message between several faces and then put it together again.

The peak signal-to-noise ratio (PSNR) is the most common metric used to evaluate the stego image quality. The PSNR measures the similarity between two images (how two images are close to each other—higher value means better results) [38]. As can be seen in Table 8, our algorithm has obtain very similar results to best state-of-the-art approaches. CC and PSNR are getting worse with decreasing quality of compression and increasing of stego message length. In terms of CC our method has overperformed [11,12] and has slightly worse results than [13,14]. In terms of PSNR our method has over performed all but [12]. We can conclude that our method is among most robust algorithms against compression attack.

## 5. Conclusions

Basing on the discussion presented in previous section we can conclude that proposed steganography method for hiding data in face images is usable and may be an interesting alternative to other state of the art approaches. The algorithm using parameters of eigenvectors linear combination turned out to be resistant to JPEG compression, clipping and scaling. These features are especially important for practical use, when facial image is only a rectangular subset of larger photo. In such cases we first need to detect the face and extract it from original image, which usually requires applying some transformations. Of course we should use the same face extraction method to generate training data set from which eigenfaces are computed. What is more, our numerical comparison with other state-of-the-art algorithms proved that eigenface-based steganography is among most robust methods against compression attack. The future work for further advancements may include improvements of robustness against downscaling attacks and taking advantage of fact that some images might contains pictures of several faces. Additional data hidden in several faces might be used as a check sum and data correction of the original secret.

## Figures and Tables

**Table 1:** This table presents values of Log-Euclidean Distance (LED), mean square error (MSE) and Pearson correlation (CC) coefficients calculated from the test data set. In order to calculate MSE and CC for each $C_{k_{a}}^{2}$ each image $I_{b}$ in training data set is encoded and then decoded using eigenfaces that explain at least 0.999 of variance.

Number of Faces	LED	Relative LED	MSE	Relative MSE	CC
2025	3452.182	–	39.092	–	0.993
4050	2463.732	0.401	29.995	0.233	0.995
6075	1337.645	0.842	24.564	0.181	0.996
8100	213.584	5.263	21.008	0.145	0.997
10,125	162.328	0.316	18.481	0.12	0.997
12,150	138.089	0.176	16.66	0.099	0.998
14,175	122.348	0.129	15.288	0.082	0.998
16,200	111.543	0.097	14.242	0.068	0.998
18,225	103.786	0.075	13.402	0.059	0.998
20,250	97.548	0.064	12.67	0.055	0.998
22,275	92.35	0.056	12.072	0.047	0.998
24,300	88.045	0.049	11.572	0.041	0.999
26,325	84.498	0.042	11.156	0.036	0.999
28,350	81.39	0.038	10.794	0.032	0.999
30,375	78.841	0.032	10.468	0.03	0.999
32,400	76.543	0.03	10.194	0.026	0.999
34,425	74.497	0.027	9.933	0.026	0.999
36,450	72.656	0.025	9.711	0.022	0.999
38,475	71.063	0.022	9.511	0.021	0.999
40,500	69.575	0.021	9.315	0.021	0.999
42,525	68.187	0.02	9.153	0.017	0.999
44,550	67.004	0.018	8.979	0.019	0.999
46,575	65.919	0.016	8.825	0.017	0.999
48,600	64.958	0.015	8.681	0.016	0.999
50,625	63.967	0.015	8.561	0.014	0.999

**Table 2:** This table presents evaluation results of robustness test of clipping (18)–(21). Values in DSC, BC and LD are averaged on whole validation data set. We have used various values of threshold parameter *t* and various lengths of hidden data.

Parameter	Length	DSC	BC	LD
0.5	18	0.001	0.079	0.167
0.6	18	0	0.012	0.022
0.7	18	0	0.001	0.001
0.8	18	0	0	0
0.9	18	0	0	0
1	18	0	0	0
0.5	37	0.003	0.295	0.896
0.6	37	0.001	0.066	0.167
0.7	37	0	0.01	0.021
0.8	37	0	0.001	0.001
0.9	37	0	0	0
1	37	0	0	0
0.5	87	0.011	0.937	7.056
0.6	87	0.004	0.616	2.622
0.7	87	0.001	0.25	0.636
0.8	87	0	0.023	0.047
0.9	87	0	0	0.001
1	87	0	0	0
0.5	174	0.02	1	23.459
0.6	174	0.008	0.985	9.991
0.7	174	0.003	0.785	3.764
0.8	174	0	0.204	0.555
0.9	174	0	0.01	0.018
1	174	0	0	0
0.5	370	0.041	1	95.991
0.6	370	0.023	1	59.254
0.7	370	0.013	1	35.393
0.8	370	0.006	0.975	15.468
0.9	370	0.001	0.424	1.834
1	370	0	0.017	0.018

**Table 3:** This table presents evaluation results of robustness test of salt and pepper. Values in DSC, BC and LD are averaged on whole validation data set. We have used various values of noise level and various lengths of encoded data.

Parameter	Length	DSC	BC	LD
1	18	0.002	0.171	0.012
2	18	0.014	0.736	0.101
3	18	0.032	0.935	0.218
4	18	0.054	0.986	0.346
1	37	0.002	0.243	0.013
2	37	0.015	0.835	0.107
3	37	0.033	0.969	0.228
4	37	0.056	0.994	0.357
1	87	0.002	0.413	0.016
2	87	0.017	0.941	0.124
3	87	0.037	0.993	0.252
4	87	0.062	0.998	0.383
1	174	0.003	0.621	0.024
2	174	0.022	0.983	0.158
3	174	0.046	0.999	0.3
4	174	0.073	1	0.435
1	370	0.009	0.891	0.065
2	370	0.043	1	0.278
3	370	0.075	1	0.44
4	370	0.107	1	0.571

**Table 4:** This table presents evaluation results of robustness test of rotation. Values in Sørensen–Dice coefficient (DSC), binary coefficient (BC) and Levenshtein distance (LD) are averaged on whole validation data set. We have used various values of rotation angle and various lengths of encoded data.

Parameter	Length	DSC	BC	LD
−1.25	18	0.182	1	0.775
−1	18	0.104	0.999	0.542
−0.75	18	0.036	0.859	0.23
−0.5	18	0.004	0.263	0.032
−0.25	18	0	0.003	0
0	18	0	0	0
0.25	18	0	0.003	0
0.5	18	0.004	0.248	0.031
0.75	18	0.035	0.819	0.218
1	18	0.1	0.992	0.521
1.25	18	0.178	1	0.761
−1.25	37	0.194	1	0.801
−1	37	0.11	1	0.572
−0.75	37	0.038	0.939	0.239
−0.5	37	0.004	0.315	0.029
−0.25	37	0	0.004	0
0	37	0	0	0
0.25	37	0	0.003	0
0.5	37	0.004	0.303	0.028
0.75	37	0.036	0.917	0.228
1	37	0.108	1	0.55
1.25	37	0.191	1	0.779
−1.25	87	0.231	1	0.864
−1	87	0.137	1	0.672
−0.75	87	0.048	1	0.306
−0.5	87	0.004	0.497	0.027
−0.25	87	0	0.003	0
0	87	0	0	0
0.25	87	0	0.003	0
0.5	87	0.004	0.531	0.027
0.75	87	0.048	1	0.299
1	87	0.143	1	0.679
1.25	87	0.24	1	0.87
−1.25	174	0.266	1	0.909
−1	174	0.169	1	0.764
−0.75	174	0.067	1	0.419
−0.5	174	0.006	0.999	0.046
−0.25	174	0	0.003	0
0	174	0	0	0
0.25	174	0	0.002	0
0.5	174	0.005	0.983	0.037
0.75	174	0.066	1	0.415
1	174	0.174	1	0.777
1.25	174	0.273	1	0.916
−1.25	370	0.341	1	0.955
−1	370	0.243	1	0.881
−0.75	370	0.121	1	0.632
−0.5	370	0.017	1	0.121
−0.25	370	0	0.001	0
0	370	0	0	0
0.25	370	0	0.001	0
0.5	370	0.015	1	0.118
0.75	370	0.122	1	0.643
1	370	0.252	1	0.892
1.25	370	0.346	1	0.961

**Table 5:** This table presents evaluation results of robustness test of JPEG compression. Values in DSC, BC and LD are averaged on whole validation data set. We have used various values of JPEG quality parameter and various lengths of encoded data.

Parameter	Length	DSC	BC	LD
95	18	0	1	0
92	18	0	1	0
89	18	0	0.999	0
86	18	0	0.973	0.002
83	18	0.002	0.828	0.012
95	37	0	1	0
92	37	0	1	0
89	37	0	0.997	0
86	37	0	0.921	0.002
83	37	0.002	0.616	0.016
95	87	0	1	0
92	87	0	1	0
89	87	0	0.959	0.001
86	87	0.001	0.642	0.007
83	87	0.004	0.22	0.032
95	174	0	1	0
92	174	0	0.985	0
89	174	0.001	0.572	0.005
86	174	0.004	0.11	0.031
83	174	0.012	0.013	0.09
95	370	0	0.996	0
92	370	0.001	0.439	0.006
89	370	0.007	0.02	0.055
86	370	0.023	0	0.161
83	370	0.045	0	0.29

**Table 6:** This table presents evaluation results of robustness test of scaling. Values in DSC, BC and LD are averaged on whole validation data set. We have used various scaling factors and various lengths of encoded data.

Parameter	Length	DSC	BC	LD
0.8	18	0.01	0.597	0.069
0.85	18	0.005	0.73	0.038
0.95	18	0.001	0.946	0.005
1	18	0	1	0
1.05	18	0	0.996	0
1.15	18	0	0.999	0
1.2	18	0	1	0
0.8	37	0.008	0.533	0.058
0.85	37	0.004	0.676	0.032
0.95	37	0	0.941	0.003
1	37	0	1	0
1.05	37	0	0.996	0
1.15	37	0	0.999	0
1.2	37	0	0.999	0
0.8	87	0.01	0.246	0.069
0.85	87	0.005	0.395	0.038
0.95	87	0	0.907	0.003
1	87	0	1	0
1.05	87	0	0.994	0
1.15	87	0	0.999	0
1.2	87	0	1	0
0.8	174	0.017	0	0.121
0.85	174	0.007	0.006	0.056
0.95	174	0	0.812	0.003
1	174	0	1	0
1.05	174	0	0.992	0
1.15	174	0	0.999	0
1.2	174	0	1	0
0.8	370	0.05	0	0.324
0.85	370	0.024	0	0.174
0.95	370	0.003	0	0.027
1	370	0	1	0
1.05	370	0	0.985	0
1.15	370	0	1	0
1.2	370	0	1	0

**Table 7:** This table presents evaluation of performance of algorithms described in Section 2.1 and Section 2.5 using various length of encoded messages and values of parameter *t*. We have calculated following statistics between original image and image with hidden data: averaged MSE, averaged maximal difference of pixels and averaged Pearson correlation coefficient of pixels.

Parameter	Length	MSE	MAX	CC
0.6	18	373.435	36.186	0.994
0.7	18	389.078	36.502	0.995
0.8	18	397.774	36.686	0.996
0.9	18	402.559	36.807	0.997
0.6	37	391.539	38.525	0.993
0.7	37	408.595	38.829	0.994
0.8	37	418.297	39.064	0.995
0.9	37	423.637	39.178	0.995
0.6	87	464.677	44.739	0.989
0.7	87	488.082	45.112	0.99
0.8	87	502.184	45.462	0.991
0.9	87	510.295	45.649	0.992
0.6	174	644.5	55.986	0.981
0.7	174	684.52	56.48	0.982
0.8	174	710.131	56.76	0.984
0.9	174	725.691	57.077	0.985

**Table 8:** This table presents comparison of Pearson correlation and peak-signal-to-noise-ratio (PSNR) for proposed and state-of-the-art methods. The number beside the proposed method (PM) is the length of the secret.

Quality of Compression	[11]	[12]	[13]	[14]	PM18	PM37	PM87	PM174
	Pearson correlation (CC)							
50	49.73	32.86	75.26	76.12	72.85	72.27	68.20	61.63
60	50.58	33.34	85.76	85.88	81.98	80.92	76.98	70.33
70	51.14	35.91	97.29	97.24	91.96	91.04	87.40	80.90
80	53.68	43.21	99.80	99.92	99.09	98.75	97.34	93.98
90	54.51	64.20	99.95	99.92	1.00	1.00	99.99	99.89
	Peak-signal-to-noise-ratio (PSNR)							
50	31.96	34.05	32.02	32.02	31.50	31.44	31.38	31.35
60	32.21	34.53	31.80	31.80	32.23	32.16	32.10	32.07
70	32.54	35.14	31.48	31.46	33.23	33.15	33.10	33.05
80	32.95	35.98	31.65	31.65	35.04	34.98	34.95	34.90
90	33.58	37.70	32.66	32.67	39.05	39.01	38.95	38.94

## Data Availability

Not applicable.
